# FEASIBILITY OF USING ASPEN BRACES TO IMPROVE POSTURE IN PISA SYNDROME ASSOCIATED WITH PARKINSON’S DISEASE: A CASE REPORT

**DOI:** 10.51894/001c.122828

**Published:** 2024-08-30

**Authors:** Cirus K. Shiran, Benjamin M, Roskiewicz, Jacek Cholewicki, Angela S. Lee, John M. Popovich Jr, John L. Goudreau

**Affiliations:** 1 College of Osteopathic Medicine Michigan State University

## 15

### INTRODUCTION

Postural deformities (i.e., Pisa syndrome, camptocormia, etc.) are common in patients with Parkinson’s disease (PD). Unfortunately, available treatment options for postural deformities in this population are limited. Although bracing has been proposed as a plausible non-invasive intervention, its adoption is impeded as PD patients with postural deformities do not tolerate bracing well due to discomfort. It is possible that a brace causes excessive involuntary muscle contractions in some PD patients, leading to muscle fatigue and discomfort. However, Aspen’s thoracolumbar braces could be more tolerable due to their unique, soft design. Therefore, the aims of this case study were to assess the effects of thoracolumbar bracing on posture and function, comfort level, quality of life (QoL), and trunk muscle activity.

### CASE DESCRIPTION

A 64-year-old woman with PD and Pisa syndrome (right lateral trunk deviation) was treated with an Aspen thoracolumbar “Peak Scoliosis Bracing System”. She was instructed to wear the brace for 4 weeks, as tolerated. To assess the effects of the brace, the following data were collected on days 0, 7, and 28: lateral trunk angle, timed up and go (TUG), postural sway (eyes open or closed and with or without the brace), comfort of the brace, QoL (EQ-5D), and trunk muscle activity (surface EMG). After four weeks of wearing the brace, there were marked improvements in the patient’s lateral trunk deviation (28 to 15 degrees) and TUG (by 4.6 seconds). Postural sway was increased when the brace was worn compared to no brace. As time progressed, the brace became more uncomfortable but ultimately corresponded to an improvement in QoL (by 0.172 in EQ index). Overall, trunk muscle activity increased by 27% when the brace was worn.

### DISCUSSION/CONCLUSION

This case study demonstrates that bracing for PD patients with Pisa syndrome might be feasible when a soft-design Aspen brace is used. The brace improved posture, function, and QoL of the patient during the 4-week period. Increased discomfort is concerning and could be linked to the increased trunk muscle activity when the brace is worn. This could also stem from overuse of the brace (6-8 hours per day) and muscle fatigue.

